# The Two-Component Model for Calculating Total Body Fat from Body Density: An Evaluation in Healthy Women before, during and after Pregnancy

**DOI:** 10.3390/nu6125888

**Published:** 2014-12-17

**Authors:** Elisabet Forsum, Pontus Henriksson, Marie Löf

**Affiliations:** 1 Department of Clinical and Experimental Medicine, Linköping University, SE 581 85 Linköping, Sweden; E-Mail: pontus.henriksson@liu.se; 2 Department of Biosciences and Nutrition, Karolinska Institute, NOVUM, SE 141 83 Huddinge, Sweden; E-Mail: marie.lof@ki.se

**Keywords:** before pregnancy, body composition, fat-free mass density, gestation, *postpartum*, total body fat

## Abstract

A possibility to assess body composition during pregnancy is often important. Estimating body density (D_B_) and use the two-component model (2CM) to calculate total body fat (TBF) represents an option. However, this approach has been insufficiently evaluated during pregnancy. We evaluated the 2CM, and estimated fat-free mass (FFM) density and variability in 17 healthy women before pregnancy, in gestational weeks 14 and 32, and 2 weeks *postpartum* based on D_B_ (underwater weighing), total body water (deuterium dilution) and body weight, assessed on these four occasions. TBF, calculated using the 2CM and published FFM density (TBF_2CM_), was compared to reference estimates obtained using the three-component model (TBF_3CM_). TBF_2CM_ minus TBF_3CM_ (mean ± 2SD) was −1.63 ± 5.67 (*p* = 0.031), −1.39 ± 7.75 (*p* = 0.16), −0.38 ± 4.44 (*p* = 0.49) and −1.39 ± 5.22 (*p* = 0.043) % before pregnancy, in gestational weeks 14 and 32 and 2 weeks *postpartum*, respectively. The effect of pregnancy on the variability of FFM density was larger in gestational week 14 than in gestational week 32. The 2CM, based on D_B_ and published FFM density, assessed body composition as accurately in gestational week 32 as in non-pregnant adults. Corresponding values in gestational week 14 were slightly less accurate than those obtained before pregnancy.

## 1. Introduction

Information regarding body composition during pregnancy is needed when estimating the requirements for dietary energy during gestation and when investigating relationships between maternal nutritional status and offspring development. Generally used body composition methods may not be appropriate during pregnancy when the body is undergoing dynamic changes to support fetal development. Based on extensive reviews of the literature Hytten [1] described how pregnancy changes the physiology of the human female including how the products of conception contribute to the composition of the pregnant body. Based on this work, van Raaij* et al.* [2] estimated average changes in fat-free mass (FFM) density and water content throughout the course of gestation. The Food and Agricultural Organization [3] and the Institute of Medicine [4] have stated that body composition methods based on these modifications [2] are satisfactory for use with pregnant women. Hopkinson* et al.* [5] evaluated these modifications in gestational week 36 and concluded that the FFM density suggested by van Raaij* et al.* [2] for women at this stage of pregnancy produce reliable mean estimates of body fat. However, except for this report [5], no validation studies have confirmed the statement quoted above [3,4].

Pregnancy is associated with retention of water, protein and mineral,* i.e.*, the components of FFM. This results in an increased water content of FFM while its concentrations of protein and mineral decrease slightly [2]. The combined effect of these changes is a decreased density of FFM [2]. This decrease is of interest when calculating total body fat (TBF) from body density (D_B_) using the so-called two-component model (2CM) [6], a common procedure for assessing human body composition* in vivo*. In the past, D_B_ was assessed using underwater weighing whereas air-displacement plethysmography (ADP) [7] is now more common. Due to its convenience and capacity to assess D_B_ in subjects with varying body sizes [7,8], ADP is increasingly used and, as pointed out by Hu [9], represents an excellent alternative to underwater weighing in pregnant women. However, the use of ADP during pregnancy has been limited [10], possibly because the capacity of the 2CM to calculate TBF from D_B_ has been insufficiently studied during gestation. Thus studies of this capacity are needed.

As indicated above, average values for FFM hydration and FFM density are available throughout the course of pregnancy [2]. However, information regarding average FFM density is insufficient to conclude that the 2CM is satisfactory during pregnancy. It is also important to determine the variability of FFM density at each particular stage of pregnancy, since small differences in this value have a substantial impact on TBF when calculated from D_B_ using the 2CM [11]. Thus a high variability in FFM density is associated with a lower accuracy in estimates of TBF of individual women. In a previous study [12] conducted before, during and after pregnancy we found that the variability of FFM hydration depends on the stage of gestation. Thus the accuracy of the 2CM may well vary during the course of pregnancy. In the present paper we use data obtained in the previous study [12] to evaluate TBF results, calculated from D_B_ using the 2CM and based on published average FFM density values. For women in the prepregnant state we used an FFM density of 1.1 g/mL [6,13]. During pregnancy, we used the values published by van Raaij* et al.* [2] and the FFM density value published by Hopkinson* et al.* [5] was used *postpartum*. We have also calculated average FFM density and its variability.

## 2. Experimental Section

### 2.1. Subjects, Design, Body Composition Methodology and Calculations

The study was conducted on 17 healthy women before pregnancy, in gestational weeks 14 and 32, and 2 weeks *postpartum*. The ethics committee in Linköping approved the study on 19 November 1995 (95237). At the measurement before pregnancy women were 29 ± 4 years old with a body mass index (BMI) of 24.3 ± 5.3 kg/m^2^. Three women (18%) were overweight (BMI = 25.0–29.9 kg/m^2^) and 2 (12%) were obese (BMI ≥ 30.0 kg/m^2^). This distribution of BMI values is similar to the corresponding distribution assessed for contemporary Swedish childbearing women [14]. All women delivered one healthy infant (birthweight 3770 ± 470 g) and none had generalized oedema. The subjects in this study, as well as the methods and procedures used were described previously [12]. In brief, D_B_ was assessed using underwater weighing where weight under water was recorded 7 times, each time with a simultaneous recording of lung volume (Volugraph 2000, Siemens-Elema, Stockholm, Sweden) [12]. Total body water (TBW) was assessed by means of deuterium dilution. After collection of background urine samples subjects received 0.05 g ^2^H_2_O per kg body weight *per os*. Five urine samples were collected during the following 15 days. ^2^H-enrichments of dose and urine samples were analyzed by using an isotope ratio mass spectrometer (Deltaplus XL, Thermoquest, Bremen, Germany) as previously described [12]. ^2^H-space was calculated using zero-time enrichment, obtained from the exponential isotope disappearance curve, providing the rate constant for ^2^H elimination. ^2^H-space was divided by 1.04 to obtain TBW [12]. D_B_ and TBW were used together with body weight to obtain reference estimates of TBF based on the three-component model (3CM) [13] where f represents the fraction of fat in the body:

f = (2.118/D_B_) − (0.78 × TBW/body weight) − 1.354 (1)

TBF was also calculated from D_B_ based on the 2CM: [6,13]

1/D_B_ = f/0.9007 + (1 − f)/FFM density (2)

FFM density was calculated using Equation (2) and f assessed by means of Equation (1).

### 2.2. Statistics

Values given are means and standard deviations (SD). Linear regression and Student’s *t* test were used. The Bland and Altman [15] procedure was used to evaluate results. Thus the mean and 2SD of the difference between TBF (%), obtained using the 3CM and TBF (%) obtained using the 2CM, were calculated. This difference (y) was regressed on the average of the two estimates of TBF (%) (x). Calculation of the methodological component of the total variability in FFM density was based on propagation of error analysis [12,16]. In this calculation precision values were 1.05% [17] for TBW and 0.01 kg [18] for body weight. Precision for D_B_ was assessed on 4 occasions in one weight-stable, non-pregnant woman with a weight and volume of 56.6 kg and 53.9 L, respectively, and was 0.0016 g/mL corresponding to 0.371 L (0.7%) for body volume [12]. As described previously [12], the methodological error was based on measurement errors expressed in two different ways,* i.e.*, in % of an appropriate mean value or in kg and L [12]. Biological variability was calculated from total observed variability and the propagated methodological error [12]. Significance was accepted when *p* < 0.05. Statistical calculations were conducted using SPSS Statistics 21 (IBM, Armonk, NY, USA).

## 3. Results

### 3.1. Total Body Fat Calculated by Means of the 2CM versus the 3CM

Table 1 shows body weight (kg), TBW (kg), D_B_ (g/mL) and TBF (%), assessed by means of the 2CM and the 3CM, of the women before pregnancy, in gestational weeks 14 and 32, and 2 weeks *postpartum*. As shown in the table, the FFM density suggested for the particular stage of reproduction was used in the 2CM. The 2CM provides lower estimates of TBF (%) than those obtained by means of the 3CM and this difference was significant before pregnancy and 2 weeks *postpartum*.

**Table 1:** Body weight, total body water, body density and total body fat assessed by means of two- and three-component models in healthy women [12] before pregnancy, at gestational weeks 14 and 32 and 2 weeks *postpartum* ^1^.

	Before Pregnancy	Gestational Week 14	Gestational Week 32	2 Weeks* Postpartum*
Body weight (kg)	66.6 ± 12.8	68.4 ± 13.2	77.3 ± 13.0	71.5 ± 12.8
Total body water (kg)	31.5 ± 4.0	32.5 ± 4.3	38.1 ± 4.4	33.6 ± 4.2
Body density (g/mL)	1.029 ± 0.019	1.027 ± 0.021	1.021 ± 0.018	1.020 ± 0.017
Total body fat (%)				
Two-component model	31.4 ± 9.0 ^2^	31.8 ± 10.1 ^3^	32.7 ± 8.8 ^4^	33.8 ± 8.5 ^5,6^
Three-component model ^7^	33.0 ± 7.9	33.2 ± 7.7	33.1 ± 7.9	35.2 ± 6.8
*p* for difference ^8^	0.031	0.16	0.49	0.043

^1^ Mean ± SD, *n* = 17; ^2^ Calculated using Equation (2) and fat-free mass density 1.1 g/mL [6,13]; ^3^ Calculated using Equation (2) and fat-free mass density 1.099 g/mL [2]; ^4^ Calculated using Equation (2) and fat-free mass density 1.092 g/mL [2]; ^5^ Calculated using Equation (2) and fat-free mass density 1.094 g/mL [5]; ^6^ The corresponding result calculated using Equation (2) and fat-free mass density 1.1 g/mL [6,13] is 35.4% ± 8.3% total body fat, which does not differ significantly from the corresponding value, assessed by means of the three-component model; ^7^ Calculated using Equation (1); ^8^ Student’s *t* test for paired observations.

**Figure 1:**
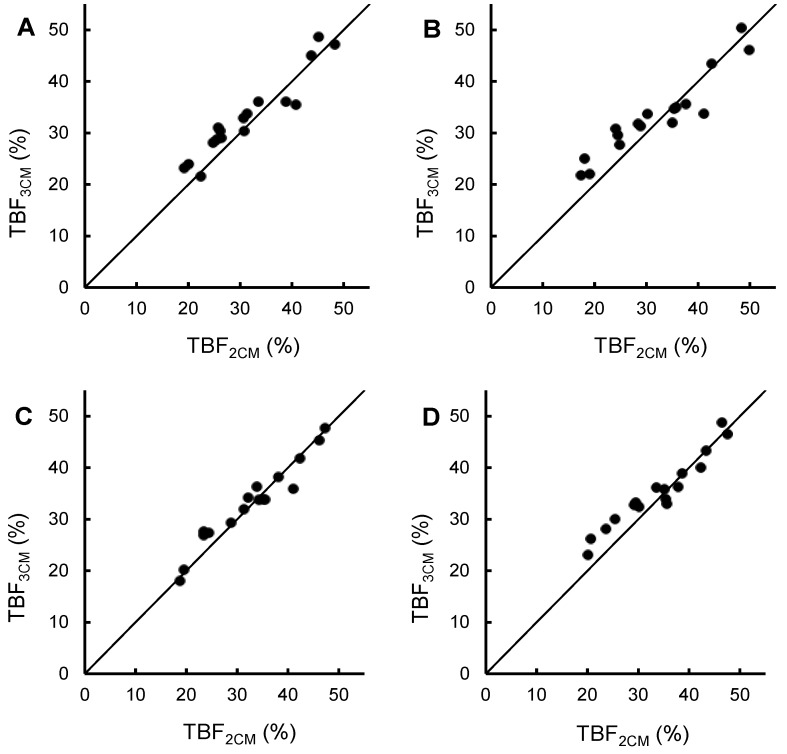
Total body fat, assessed using a three-component model (*y*)* versus* total body fat, assessed using a two-component model (*x*) in 17 healthy women [12] plotted around the line of identity. The fat-free mass density used in the two-component model is also given. (**A**) Before pregnancy, FFM density = 1.1 g/mL [6,13]; (**B**) Gestational week 14, FFM density = 1.099 g/mL [2]; (**C**) Gestational week 32, FFM density = 1.092 g/mL [2]; (**D**) 2 weeks *postpartum*, FFM density = 1.094 g/mL [5]. FFM, fat-free mass; TBF_3CM_, total body fat assessed using a three-component model; TBF_2CM_, total body fat, assessed using a two-component model.

Figure 1 shows TBF (%) of the women in the study, obtained by means of the 2CM (*x*) and the 3CM (*y*), and plotted around the line of identity. The regression equations were *y* = 0.84*x* + 6.8, *r* = 0.95, *p* < 0.001 (before pregnancy), *y* = 0.72*x* + 10.3, *r* = 0.94, *p* < 0.001 (gestational week 14), *y* = 0.87*x* + 4.5, *r* = 0.97, *p* < 0.001 (gestational week 32) and *y* = 0.78*x* + 8.9, *r* = 0.96, *p* < 0.001 (2 weeks *postpartum*). Figure 1 demonstrates that the 2CM and the 3CM agree fairly well on all measurement occasions.

**Figure 2:**
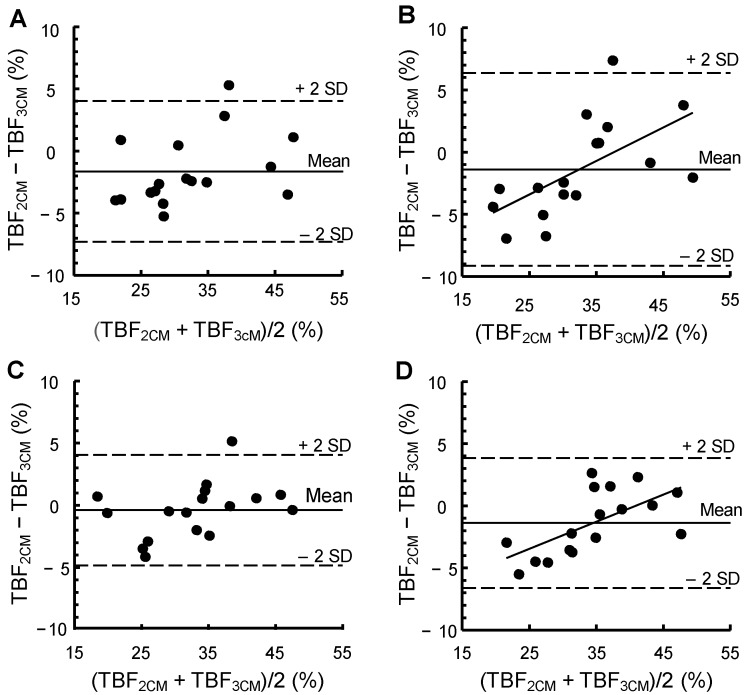
A Bland and Altman evaluation of a two-component model ^1^, based on body density, for assessing TBF (%) in women before pregnancy, at gestational weeks 14 and 32 and 2 weeks *postpartum* when compared to reference estimates of TBF (%) obtained by means of a three-component model ^2^. The figure shows the average and 2SD of the difference between the two estimates and the correlation coefficient (*r*) and *p*-value for the relationship obtained when (TBF_2CM_ − TBF_3CM_) (%) (*y*) is regressed on (TBF_2CM_ + TBF_3CM_)/2 (%) (*x*). When *r* is significant (*p* < 0.05), the regression equation for this relationship is given. (**A**) Before pregnancy, (TBF_2CM_ − TBF_3CM_) = −1.63%, 2SD = 5.67%, *r* = 0.39 (*p* = 0.12); (**B**) Gestational week 14, (TBF_2CM_ − TBF_3CM_) = −1.39%, 2SD = 7.75%, *r* = 0.61 (*p* = 0.009), *y* = 0.27*x* − 10.2; (**C**) Gestational week 32, (TBF_2CM_ − TBF_3CM_) = −0.38%, 2SD = 4.44%, *r* = 0.40 (*p* = 0.12); (**D**) 2 weeks *postpartum*: (TBF_2CM_ − TBF_3CM_) = −1.39%, 2SD = 5.22%, *r* = 0.63 (*p* = 0.006), *y* = 0.22*x* − 8.9. Using Equation (2) and the fat-free mass density 1.1 g/mL [6,13]: (TBF_2CM_ − TBF_3CM_) = −0.25%, 2SD = 5.93%, *r* = 0.59 (*p* = 0.014), *y* = 0.19*x* − 6.6. TBF, total body fat; TBF_2CM_, total body fat assessed using the two-component model; TBF_3CM_, total body fat assessed using the three-component model.

^1^ The two-component model is based on Equation (2) and the fat-free mass density 1.1 g/mL (before pregnancy) [6,13], 1.099 g/mL (gestational week 14) [2], 1.092 g/mL (gestational week 32) [2] and 1.094 g/mL (2 weeks *postpartum*) [5]; ^2^* n* = 17.

Figure 2 shows a Bland and Altman evaluation of our data. 2SD corresponds to 7.75% and 4.44% TBF at gestational weeks 14 and 32, respectively. The corresponding values before pregnancy and 2 weeks *postpartum* were 5.67% and 5.22% TBF, respectively. At gestational week 14 and 2 weeks *postpartum*, significant relationships were found between TBF (%), assessed by means of the 2CM minus TBF (%) assessed by means of the 3CM, on the one hand, and the average of these two estimates, on the other hand. The corresponding relationships before pregnancy and in gestational week 32 were not significant.

### 3.2. FFM Density and Its Variability

Table 2 shows estimated average FFM density and its variability before pregnancy, at gestational weeks 14 and 32, and 2 weeks *postpartum*. FFM density was 1.106, 1.104, 1.093 and 1.099 g/mL, before pregnancy, at gestational weeks 14 and 32, and 2 weeks postpartum, respectively. No significant differences between these values and the corresponding values published by van Raaij* et al.* [2] were observed at gestational weeks 14 or 32. Table 2 also shows the biological variability of FFM density, estimated on the same occasions and based on the two sets of methodological errors. These two sets are described in footnotes 3 and 4 of Table 2. Before pregnancy, biological variability was 0.007 and 0.009 g/mL when using the first and second sets of assumptions, respectively. The impact of pregnancy on the biological variability of FFM density was smaller in gestational week 32 than in gestational week 14, and this was the case for both sets of assumptions.

## 4. Discussion

The 2CM is considered to be capable of generating useful body composition results in healthy adult subjects in the general population if a valid figure for FFM density is used [7]. The data in Figure 1 show that there is considerable agreement between the 2CM and the 3CM regarding their capacity to assess TBF (%) before, during and after pregnancy, although the results of the two models may differ for individual women. Thus our data support previous statements [3,4] that the 2CM, based on appropriate FFM density values, can provide useful body composition results also during pregnancy. However, as discussed below, the accuracy of such results may vary during pregnancy.

In this paper, we use a 3CM to obtain reference estimates of TBF before, during and after pregnancy, although a four-component model (4CM) is generally regarded as the best option when evaluating simpler body composition methods [5,6]. A commonly used 4CM calculates TBF from body weight, TBW, D_B_ and body mineral and has the advantage over a 3CM of avoiding assumptions about the mineral content of the body [6]. However, the theoretical errors in estimates of TBF (%), associated with variations in FFM composition, are not very different between the 4CM and the 3CM [11]. This can be reconciled with results in women showing that the water content of FFM was significantly related to the difference in TBF (%) assessed by means of a 2CM* versus* a 4CM, while the corresponding relationship for the mineral content of FFM was weaker and not significant [19]. Thus a 3CM, based on TBW, D_B_ and body weight can provide useful reference estimates of body composition in healthy women.

Application of a 4CM in pregnant women is not regarded as appropriate since an assessment of body mineral requires the use of dual-energy X-ray absorptiometry, a technique that is unacceptable for such subjects due to radiation exposure. The body mineral content increases during gestation due to the contribution of the fetus. In gestational week 32, the fetus increases the contents of osseous and non-osseous minerals in the pregnant body, by about 1% each [6,20,21]. Such changes have only a minor impact on TBF (%), calculated by means of the 3CM, since Elia estimated that increases in the body mineral content as high as 20%–25% affect such estimates by less than one unit TBF (%) [11]. Furthermore, for women in gestational week 36, Hopkinson* et al.* [5] compared the 3CM to the 4CM and found the results to be in close agreement. In this comparison, the body mineral content required by the 4CM was measured 2 weeks postpartum and the authors [5] reckoned that the magnitude of changes in this variable between week 36 of gestation and 2 weeks postpartum was too small to have any appreciable effect on the results. Based on this evidence, we conclude that the 3CM provides useful reference estimates of body composition also in pregnant women.

**Table 2:** Average and total variability of FFM density in women before, during and after pregnancy. The contributions to total variability of methodological error and biological variability using two sets of assumptions are also shown.

	Average FFM density ^1^g/mL	Total variability of FFM density	Propagation of error analysis 1 ^2^				Propagation of error analysis 2 ^3^			
			Methodological error		Biological variability		Methodological error		Biological variability	
		SD	SD	% ^4^	SD	% ^4^	SD	% ^4^	SD	% ^4^
Before pregnancy	1.106	0.010	0.007	49	0.007	51	0.006	33	0.009	67
Gestational week 14	1.104	0.014	0.007	28	0.012	72	0.006	18	0.013	82
Gestational week 32	1.093	0.008	0.007	96	0.002	4	0.005	47	0.006	53
2 weeks *postpartum*	1.099	0.009	0.007	70	0.005	30	0.006	41	0.007	59

^1^ Calculated using Equation (2) and a fraction of fat in the body (f) calculated using Equation (1). ^2^ Errors for total body water and body volume are expressed in % of an appropriate mean value. The calculations are based on the following precision values [12]: body weight 0.01 kg; total body water 1.05% corresponding to 0.331 kg in women before pregnancy, 0.341 kg in gestational week 14, 0.400 kg in gestational week 32, and 0.352 kg 2 weeks *postpartum*; body volume 0.7% corresponding to 0.455 L in women before pregnancy, 0.467 L in gestational week 14, 0.531 L in gestational week 32, and 0.492 L 2 weeks *postpartum*. ^3^ Errors for total body water and body volume are expressed in kg and L, respectively. The calculations are based on the following precision values [12]: 0.01 kg for body weight; 0.331 L for total body water and 0.371 L for body volume. ^4^ Percentage of total variability. *n* = 17; FFM, fat-free mass.

Our estimated FFM density of women prior to pregnancy is in agreement with the figure, 1.1 g/mL, which is the value generally regarded as an appropriate average for the general population [7]. However, it may be relevant to note that the 2CM underestimated TBF of our women before pregnancy when this FFM density was used and Fields* et al.* [19] reported a similar underestimate by the 2CM in their study on healthy women also using this FFM density. This suggests that women may have a slightly higher FFM density than 1.100 g/mL, which is the value for the reference body [6]. However, confirming this suggestion is difficult, since available techniques for assessing FFM density* in vivo* may not be accurate enough. Small variations in this variable between different populations of women may also be present.

We estimated average FFM density 2 weeks *postpartum* at 1.099 g/mL, which is slightly higher than 1.094 g/mL as reported by Hopkinson* et al.* [5]. A consequence of using this lower value is that the 2CM underestimates TBF (%) *postpartum*. Furthermore, as shown by the Bland and Altman evaluation, the magnitude of the difference between the two models is associated with TBF (%) of the women. Using an FFM density of 1.1 g/mL 2 weeks *postpartum* produces more satisfactory average estimates of TBF (%), but, as indicated in Figure 2, the difference between the models regarding TBF (%) is still associated with TBF (%) of the women.

Our results regarding the biological variability of FFM density were obtained using two sets of methodological errors. As previously discussed [12] we found both sets to be reasonable and justified and we were not able to determine whether one was superior to the other. Prior to pregnancy, we found the biological variability of FFM density to be 0.007 and 0.009 g/mL, respectively, using the two sets of methodological errors. This is comparable to the published values of 0.0084 g/mL [6] and 0.0073 g/mL [22]. This rather large biological variability of FFM density has a negative impact on the accuracy of the 2CM in non-pregnant women. The finding that the biological variability in FFM density is lower at gestational week 32 but higher at gestational week 14 when compared to the value before pregnancy is a corollary of our previous findings [12] regarding the biological variability of FFM hydration at gestational weeks 14 and 32* versus* before pregnancy. These observations are consistent with data in Figure 2 showing that the 2SD value at gestational week 32 is only 4.44% TBF as opposed to 5.67% and 7.75% TBF before pregnancy and at gestational week 14, respectively. These observations indicate that the accuracy of the 2CM, when based on published FFM density values [2], is better at gestational week 32 than at gestational week 14.

Published values for FFM density in pregnant women [2] are based on data by Hytten [1], collected in British women several decades ago. Nevertheless, the differences between these values [2] and our estimates of FFM density during pregnancy were small and not significant. This supports the conclusion that the FFM density values by van Raaij* et al.* [2] produce valid average estimates of TBF of women when used in a 2CM at gestational weeks 14 and 32. Furthermore, we suggest that these observations can be reconciled with the statement that the influence of pregnancy on FFM density and composition is predictable and part of the regulatory processes needed to meet the physiological changes inherent in this physiological state.

A limitation of this study is that the number of women is small, while a strength is that the same women were studied before, during and after pregnancy. This made it possible to demonstrate how pregnancy influences FFM density and its variability which is important information for assessing body composition during pregnancy. It is also relevant to emphasize that our results were obtained in healthy women being pregnant with one fetus and may not be valid for women not meeting these criteria. Furthermore, our results were obtained in women with a BMI distribution typical for Swedish women [14] and may not be appropriate in populations with a different pattern of body weight.

## 5. Conclusions

This study confirmed that FFM density values at gestational weeks 14 and 32 are in close agreement with those published by van Raaij* et al.* [2]. We found the corresponding value 2 weeks *postpartum* to be close to 1.1 g/mL which is the figure commonly regarded as an appropriate average for healthy non-pregnant adults. Furthermore, our results showed that a 2CM, based on estimates of D_B_ and the FFM density values reported by van Raaij* et al.* [2], is able to generate body composition results in gestational week 32 that are at least as accurate as those obtained in the pre-pregnant state with the same methodology when using a FFM density of 1.1 g/mL. Corresponding values obtained in gestational week 14 were found to be slightly less accurate than those obtained before pregnancy.
